# Knowledge, attitudes, and practices [KAP] toward COVID-19: A cross-sectional study in the New York Metropolitan Area and California Bay Area

**DOI:** 10.1371/journal.pone.0271212

**Published:** 2022-08-10

**Authors:** Erica Mark, Galina Udod, Jayne Skinner, Marieke Jones

**Affiliations:** 1 University of Virginia School of Medicine, Charlottesville, Virginia, United States of America; 2 Downstate College of Medicine, State University of New York, Brooklyn, New York, United States of America; 3 Department of Medical Sciences, Brown University, Providence, Rhode Island, United States of America; 4 Claude Moore Health Sciences Library, University of Virginia, Charlottesville, Virginia, United States of America; VIT University, INDIA

## Abstract

**Objective:**

The 2019 novel coronavirus [COVID-19] pandemic has necessitated the implementation of public health initiatives [PHI] to slow viral spread. We evaluated the effectiveness of PHI through a survey of COVID-19 knowledge, attitudes and practices [KAP].

**Methods:**

This cross-sectional study was conducted primarily during stay-at-home orders in New York and San Francisco. A volunteer sample of 675 U.S. participants completed a KAP questionnaire after electronic distribution.

**Results:**

Participants had good knowledge and practices, but poor attitudes. Predictors of higher knowledge scores included white ethnicity, non-essential worker status, and healthcare worker status. Correlates with positive attitude included male gender, residence in California, higher annual income, and not utilizing radio or social media. Higher practice scores were predicted by female gender, non-essential and healthcare worker status, and information source.

**Conclusions:**

Differences in KAP were found among demographic variables. Determining what factors and sources of information drive reception of public health information can guide targeted intervention and advance equitable health education.

## Introduction

The 2019 novel coronavirus [COVID-19] reached pandemic proportions following identification in December 2019 in Wuhan, China [1]. The current pandemic has challenged public health organizations and governments, overwhelming healthcare systems. In response, many countries have implemented measures to slow the spread of the virus and prevent unnecessary deaths. These efforts have been proven to be effective in curtailing the spread of disease [2, 3].

An effective public health education program is essential to disease prevention [4]. The goal of this education is not only to reduce the number of individuals affected with the virus, but also to reduce the overall burden on our healthcare system’s resources and avoid preventable deaths. To determine the effectiveness of public health strategies, it is pertinent to evaluate whether the population has accurately received knowledge about public health practices and how the public’s general attitudes and beliefs affect behavior [5]. Compliance with health protective behaviors is essential and is influenced by knowledge, attitudes, and practices [KAP] regarding COVID-19 [6, 7]. Data from the 2002 SARS and 2012 MERS outbreaks support that KAP toward disease outbreaks affect individual action [8–14]. Furthermore, disparities in COVID-19 knowledge have been associated with socioeconomic patterns, with undereducated individuals and lower income households more likely to underestimate risks associated with COVID-19 and have less knowledge of disease symptoms [15, 16].

Receptiveness to disease prevention measures and knowledge of the severity of the disease are vital in understanding the impact of public health campaigns as risk perception significantly influences precautions taken [11, 14, 17]. Due to the novelty of the virus, information regarding transmission, treatment and prevention has not been clear and easily accessible. The lack of coherent guidelines and vague sometimes contradictory messaging from political figures has created an atmosphere for misinformation to spread [18, 19]. Accordingly, people may choose to not adhere to beneficial public health practices when presented with contradictory misinformation [5]. Furthermore, disparities in COVID-19 knowledge have been associated with socioeconomic patterns; undereducated individuals and lower income households have been shown to be more likely to underestimate risks associated with COVID-19 and have less knowledge of disease symptoms [20–22].

The United States was unique in leading the world in both caseload and fatalities prior to vaccine distribution. The San Francisco Bay Area was the first in the country to issue a shelter in place order beginning on March 16, 2020 after 258 confirmed coronavirus cases and three deaths. On March 22, 2020, a stay-at-home order was issued in New York City [23]. In the following months, San Francisco had over 4,000 confirmed cases and 50 deaths. In contrast, New York had over 225,000 confirmed cases and just under 23,000 deaths as of July 16, 2020 [24]. Investigating KAP differences in California and New York during the early months of the pandemic can provide potential stakeholders such as public health policymakers and healthcare workers with an understanding of KAP deficiencies associated with specific demographics and inform public health education strategies to reduce the disproportionate impact of COVID-19 on vulnerable groups.

Survey data should be collected on public perception of COVID-19 to support efforts to mitigate outbreak. Since the onset of the pandemic, there have been ongoing KAP reports across the United States from the Johns Hopkins Center for Communication Programs [25]. However, this study will be the first cross-sectional KAP analysis of California and New York to evaluate the effectiveness and potential shortcomings of public health education. Our study suggests that interpretation and implementation of health education varies by race, gender, income, employment, and information source. Public health education is often the first-line of defense in disease prevention. This research is important to minimize health inequality and ensure that this education is received equally by all subsets of the population.

## Methods

### Design

This was a non-experimental, cross-sectional study. The New York Metropolitan Area and the San Francisco Bay Area were chosen due to high COVID-19 caseload, diverse populations, and ample evidence of local public health organization involvement. In-person restrictions necessitated online survey administration.

### Participants

Volunteer participants were recruited via social media. Community leaders in areas of local government, religious institutions, and community service organizations were contacted and asked to distribute the survey to their mailing lists. Additionally, the survey was posted on all publicly available Facebook groups using location specific search terms until saturation: San Francisco, Bay Area, Alameda, Contra Costa, Marin, Napa, San Mateo, Santa Clara, Solano, Sonoma, New York City, Bronx, Brooklyn, Manhattan, Queens, Staten Island, New Jersey, and Hudson Valley. Participants represent a diverse, cross-sectional sample of individuals from multiple different interest groups, and are likely an adequate sample of the U.S. population at large. The recruitment flyer included a summary of the study, time required, emphasis on the voluntary and confidential nature of participation, a point of contact for questions, and a link and QR code. Although the study aimed to recruit participants in the aforementioned areas, participants located in other areas were not excluded from completing the survey. Participants younger than 18 were excluded.

### Questionnaire

The 92-item survey was developed with the assistance of an academic psychiatrist, emergency medicine physician, and statistician using an iterative process [S1 Appendix]. Cronbach’s alpha, a measure of internal consistency, was 0.730 for knowledge, 0.687 for attitude, 0.629 for practices with values > 0.6 considered satisfactory. The scoring system is featured in S2 Appendix.

### Data collection

Institutional review board approval was obtained from the University of Virginia. Data were collected between 4/20/20 and 5/22/20 via Qualtrics. No financial incentive was provided. The survey received 776 responses and 101 participants were excluded based on incomplete data or international residence. Therefore, 675 responses were used for data analysis. Our targeted sample size was determined from a literature review of KAP studies regarding infectious diseases, which had sample sizes ranging from 85–1,147 with a mean of 479 and a median of 373 [6–14].

### Data analysis

Data were cleaned and exported to SPSS 26.0 software^®^ [IBM Corporation, Armonk, NY, USA]. Equal variance ANOVAs were conducted to analyze differences in demographic variables for KAP scores in order to select variables for inclusion in the multivariate regression models [S3 Appendix].

Three multivariable linear regression models were created using the demographic variables selected in the univariate ANOVAs as independent variables and KAP scores as dependent variables. Assumptions were checked on the three final multivariate regression models using QQ plots [to assess for normality of residuals], plots of residuals against predicted values [to assess for equal variance, linearity, and no other systematic patterns], and plots of residuals against excluded predictors [to ensure residuals solely represent random noise].

Individual questions as indicated in S2 Appendix were not included in the final analysis. In analyzing marital status, education, household size, and housing type, categories with small numbers of participants [< 10 participants] were removed or regrouped as indicated in the tables. Factors were selected in a backward stepwise method using adjusted R^2^ as the model fit criteria. Unstandardized regression coefficients [β] and their 95% confidence intervals were used to quantify the association between demographic variables and KAP. Assumptions were checked on the three final multivariate regression models and were adequately met in all three models. The significance threshold was set at p < 0.05.

Pearson correlation was performed to assess the strength of the relationship between each pair of KAP categories.

## Results

### Social and demographic characteristics

Table 1 includes the demographic characteristics of our study sample. Out of 675 participants, 480 [71.1%] identified as female, 162 [24.0%] as male, and 33 [4.9%] as non-binary. Most participants indicated they are in the 25–39 [209. 30.96%] or 40–55 [181, 26.81%] age groups. Respondents were primarily residents of California [365, 54.07%] and New York [207, 30.67%], and 103 [15, 26%] claimed another state as their current residence. Additionally, 139 participants [20.59%] identified as an essential worker, and 98 participants [14.52%] as a healthcare worker. The majority listed the internet and social media as one of their main sources of information [558, 82.22%] and only 70 [10.37%] listed that they received information about COVID-19 from a healthcare worker.

**Table 1:** Demographic statistics of participants and KAP values.

Variable	Mean	SD	Min	Max	N	%
Knowledge Score	93.16	6.75	14.86	100		
Attitude Score	57.11	9.57	25.75	84.77		
Practice Score	89.72	9.32	34	100		
**Gender**						
He/him/his					162	24.00
She/her/hers					480	71.11
They/them/theirs					32	4.74
Ze/hir					1	0.15
**Age**						
18–24					156	23.11
25–39					209	30.96
40–59					181	26.81
60 and over					129	19.11
**Marital Status**						
Single					205	30.37
In a relationship					156	23.11
Married					248	36.74
Divorced					49	7.26
Widowed					9	1.33
Prefer not to say					8	1.19
**Education**						
Middle school					1	0.15
High school					109	16.15
College					345	51.11
Graduate school					220	32.59
**Income**						
Less than $15,000					96	14.22
$15,000–34,999					70	10.37
$35,000–49,999					73	10.81
$50,000–74,999					115	17.04
$75,000–150,000					154	22.81
Greater than $150,000					89	13.19
Prefer not to say					78	11.56
**Race**						
Caucasian					507	67.33
Hispanic or Latino					59	7.84
African American					30	3.98
Asian					94	12.48
Native American					11	1.46
Other					41	5.44
Prefer not to say					11	1.46
**Household Size**						
Just me					112	16.59
1–3					375	55.56
4–5					165	24.44
6 or more					23	3.41
**Community Type**						
Rural					38	5.63
Suburban					278	41.19
Urban					359	53.19
**State**						
California					365	54.07
New York					207	30.67
Other					103	15.26
**Housing Type**						
A house					382	56.59
An apartment					282	41.78
Other					11	1.63
**Employment Status**						
Yes					436	64.59
No					239	35.41
**Student**						
Yes					169	25.04
No					506	74.96
**Essential Worker**						
Yes					139	20.59
No					536	79.41
**Healthcare Worker**						
Yes					98	14.52
No					577	85.48
**Information Source**						
Radio					122	6.64
Newspaper					271	14.76
Scholarly article					198	10.78
Television					309	16.83
Neighbors and friends					163	8.88
Internet and social media					555	30.23
Doctor					83	4.52
Coworker					33	1.80
Healthcare worker					70	3.81
Other					32	1.74
**COVID-19 Status**						
I have tested negative for COVID-19					37	5.48
I have tested positive for COVID-19					12	1.78
I have not been tested for COVID-19					626	92.74
**Affiliation With COVID-19 Patient**						
Has COVID-19					294	43.56
Is in the hospital with COVID-19					62	9.19
I do not know anyone with COVID-19					319	47.26

### Overall scores

Participants overall had good knowledge [93.16 ± 6.75] and practices [89.72 ± 9.32], defined at > 80%, but poor attitude [57.11 ± 9.57]. Pearson correlations demonstrated a weak positive correlation between knowledge and attitude [r = 0.134, p < 0.001] and between knowledge and practices [r = 0.190, p < 0.001]. No correlation was found between attitude and practices [r = 0.057, p = 0.141] [Table 2].

**Table 2:** Pearson correlations between KAP.

	Attitude		Practice	
	Pearson Correlation	P	Pearson Correlation	P
Knowledge	0.134	<0.001	0.190	<0.001
Attitude			0.057	0.141

### Multivariate regression

The multivariate regression models are found in Table 3.

**Table 3:** KAP multivariate regressions.

**Result of Multiple Regression Analysis on Factors Significantly Associaited With Higher COVID-19 Knowledge Scores (*R***^***2***^ ***= 0*.*156*, *F = 5*.*78*, *df = 26*, *p<0*.*001)***							
		Unstandardized coefficients		Standardized Coefficients		Confidence Statistics	
**Model**		**B**	**Std Error**	**T (df)**	**Sig**	**Lower Bound**	**Upper bound**
Intercept		94.56	3.58	26.42 (1)	0.000	87.54	101.59
Gender	Non-binary vs Female	-2.98	1.13	-2.63 (2)	0.009	-5.20	-0.75
	Male vs Female	-0.73	0.58	-1.27 (2)	0.204	-1.87	0.40
Essential Worker	Yes vs No	-1.49	0.64	-2.32 (1)	0.021	-2.74	-0.23
Healthcare Worker	Yes vs No	2.29	0.74	3.08 (1)	0.002	0.83	3.74
Source of information (not utilizing ___ vs utilizing ____)	Newspaper	-2.29	0.62	-3.71 (1)	0.000	-3.50	-1.08
	Radio	-0.21	0.72	-0.29 (1)	0.771	-1.62	1.20
	Healthcare worker	-0.11	0.87	-0.13 (1)	0.899	-1.83	1.60
	Coworker	1.05	1.17	0.90 (1)	0.370	-1.25	3.35
	Internet or Social Media	-1.12	0.80	-1.41 (1)	0.159	-2.69	0.44
	Scholarly Articles	-1.17	0.66	-1.77 (1)	0.077	-2.47	0.13
	Friends/Neighbors	-0.69	0.68	-1.02 (1)	0.307	-2.02	0.64
	Television	0.37	0.61	0.61 (1)	0.544	-0.83	1.57
	Doctor	-0.76	0.83	-0.92 (1)	0.356	-2.38	0.86
	Other	2.71	1.22	2.22 (1)	0.027	0.31	5.10
Race	Hispanic vs Caucasian	-4.91	1.23	-4.00 (5)	0.000	-7.33	-2.50
	Asian vs Caucasian	-2.97	0.79	-3.77 (5)	0.000	-4.52	-1.43
	Black vs Caucasian	-4.73	1.38	-3.44 (5)	0.001	-7.43	-2.03
	Prefer not to say vs Caucasian	-3.30	1.92	-1.72 (5)	0.086	-7.07	0.46
	Multiracial vs Caucasian	-3.14	0.76	-4.14 (5)	0.000	-4.63	-1.65
Location	Urban vs Suburban	0.05	0.53	0.09 (2)	0.930	-1.00	1.10
	Rural vs Suburban	-2.27	1.12	-2.03 (2)	0.043	-4.46	-0.08
Level of Education	Graduate School vs < college	1.45	0.82	1.761 (2)	0.079	-0.17	3.06
	College vs < college	0.86	0.73	1.19 (2)	0.234	-0.56	2.29
Current student	Yes vs No	-0.11	0.63	-0.18 (1)	0.856	-1.35	1.13
State	Other states vs California	-0.09	0.72	-0.12 (2)	0.902	-1.50	1.32
	NY vs California	-2.00	0.57	-3.48 (2)	0.001	-3.13	-0.87
**Results of Multiple Regression Analysis for Factors Significantly Associated With Higher COVID-19 Attitude Scores *(R***^***2***^ ***= 0*.*059*, *F = 2*.*32*, *df = 32*, *p<0*.*001*)**							
	Intercept	45.22	6.53	6.92 (1)	0.000	32.39	58.05
Income level	> $150,000 vs < $15,000	4.60	1.69	2.72 (6)	0.007	1.27	7.93
	$15,000-$34,999 vs < $15,000	1.48	1.59	0.93 (6)	0.351	-1.64	4.61
	$35,000 -$49,999 vs < $15,000	2.71	1.60	1.70 (6)	0.090	-0.42	5.85
	$50,000 -$74,999 vs < $15,000	2.64	1.58	1.67 (6)	0.096	-0.47	5.73
	$75,000 - $150,000 vs < $15,000	2.29	1.56	1.47 (6)	0.141	-0.76	5.35
Location	Urban vs Suburban	0.39	0.87	0.44 (2)	0.657	-1.33	2.10
	Rural vs Suburban	-0.15	1.69	-0.09 (2)	0.928	-3.47	3.17
Living arrangement	Apartment vs House	4.73	2.91	1.63 (2)	0.104	-0.98	10.44
	Other vs House	3.42	2.94	1.16 (2)	0.245	-2.36	9.20
COVID-19 Status	No COVID-19 test vs tested positive	-2.52	2.80	-0.90 (2)	0.367	-8.02	2.97
	Tested negative vs tested positive	-5.67	3.15	-1.80 (2)	0.072	-11.86	0.51
Current Student	Yes vs No	1.36	1.18	1.15 (1)	0.251	-0.96	3.68
Gender	Non-binary vs Females	-2.66	1.71	-1.55 (2)	0.121	-6.03	0.70
	Males vs Females	2.57	0.87	2.96 (2)	0.003	0.87	4.28
Healthcare Worker Status	Yes vs No	1.57	1.11	1.42 (1)	0.157	-0.61	3.75
Essential Worker Status	Yes vs No	-0.99	0.99	-1.00 (1)	0.318	-2.92	0.95
Age	[Age_Coded = 1]	1.96	1.44	1.362	0.174	-0.87	4.79
	[Age_Coded = 2]	0.30	1.33	0.228	0.820	-2.30	2.91
	[Age_Coded = 3]	0.10	1.15	0.089	0.929	-2.16	2.37
State	New York vs California	-2.22	0.90	-2.46 (2)	0.014	-3.99	-0.44
	Other vs California	-0.84	1.08	-0.77 (2)	0.440	-2.96	1.29
Source of information (Not utilizing ___ vs utilizing ____)	Radio	2.89	1.08	2.67 (1)	0.008	0.76	5.01
	Doctor	0.00	1.26	-0.00 (1)	0.997	-2.48	2.47
	Healthcare worker	0.93	1.31	0.71 (1)	0.475	-1.63	3.49
	Television	0.41	0.91	0.46 (1)	0.649	-1.37	2.20
	Newspaper	-0.05	0.92	-0.06 (1)	0.956	-1.86	1.76
	Coworker	1.28	1.76	0.73 (1)	0.467	-2.17	4.72
	Friend/Neighbor	0.88	1.02	0.86 (1)	0.393	-1.13	2.88
	Internet and social media	2.95	1.20	2.46 (1)	0.014	0.59	5.31
**Results of Multiple Regression Analysis For Factors Significantly Associated With Worse COVID-19 Practice Scores *(R***^***2***^ ***= 0*.*199*, *F = 8*.*17*, *df = 23*, *p< 0*.*001)***							
	Intercept	103.81	4.84	21.44 (1)	0.000	94.30	113.31
Gender	Males vs Females	-3.54	0.82	-4.32 (2)	0.000	-5.15	-1.93
	Non-binary vs Females	-1.57	1.59	-0.99 (2)	0.322	-4.69	1.54
Location	Rural vs Suburbs	-4.55	1.58	-2.89 (2)	0.004	-7.64	-1.46
Healthcare Worker Status	Yes vs No	-0.21	1.04	-0.21 (1)	0.837	-2.25	1.83
Essential worker status	Yes vs No	-3.15	0.91	-3.45 (1)	0.001	-4.95	-1.36
Source of information about COVID-19 (Not utilizing _____ vs utilizing)	Newspapers	-3.14	0.85	-3.67 (1)	0.000	-4.81	-1.46
	Coworker	0.64	1.63	0.40 (1)	0.693	-2.55	3.84
	Other	-1.91	1.69	-1.13 (1)	0.259	-5.23	1.41
	Internet and social media	-1.23	1.12	-1.09 (1)	0.275	-3.43	0.98
	Friend/neighbor	-0.25	0.94	-0.27 (1)	0.788	-2.10	1.59
	Doctor	-1.88	1.16	-1.61 (1)	0.107	-4.16	0.41
	Healthcare worker	-2.51	1.22	-2.06 (1)	0.040	-4.90	-0.12
	Scholarly articles	-2.88	0.92	-3.14 (1)	0.002	-4.68	-1.08
	Television	-2.34	0.84	-2.78 (1)	0.006	-3.99	-0.69
Housing type	Alternative living arrangement vs House	-5.72	2.69	-2.12 (2)	0.034	-11.00	-0.43
	Apartment vs House	-2.26	0.84	-2.69 (2)	0.007	-3.91	-0.61
Marital Status	In a Relationship vs Single	-7.59	1.00	-7.60 (3)	0.000	-9.55	-5.63
	Married vs Single	-6.03	0.90	-6.68 (3)	0.000	-7.81	-4.26
	Divorced/widowed vs Single	0.93	1.34	0.697 (3)	0.486	-1.70	3.56
State	New York vs California	0.69	0.88	0.79 (2)	0.432	-1.03	2.41
	Other vs California	-1.16	1.02	-1.13 (2)	0.259	-3.16	0.85

### Knowledge

The multivariate regression analysis showed that knowledge differed by race, specifically that Hispanic [β = -4.914, t_5_ = -3.998, p < 0.001], Asian [β = -2.972, t_5_ = -3.774, p < 0.001], African American [β = -4.729, t_5_ = -3.44, p = 0.001] and multiracial respondents [β = -3.14, t_5_ = - 4.138, p < 0.001] had lower knowledge scores compared to White respondents. Non-essential workers had greater knowledge scores than essential workers [β = -1.485, t_1_ = -2.32, *p* = 0.021], and healthcare workers had greater knowledge scores than non-healthcare workers [β = 2.287, t_5_ = 3.081, *p* = 0.002]. Participants in New York scored lower on knowledge than those in California [β = -2, t_2_ = -3.481, *p* = 0.001] but not different from participants in other states [β = -0.089, t_2_ = -0.124, *p* = 0.902]. The adjusted R^2^ for the model was 15.6% [p < 0.001].

### Attitudes

Males had higher attitude scores than females [β = 2.572, t_2_ = 2.964, *p* = 0.003] but not different than non-binary participants [β = -2.66, t_2_ = -1.553, *p* = 0.121]. New York residence predicted lower attitude scores compared to California [β = -2.217, t_2_ = -2.455, p = 0.014] but there was no difference in attitude compared to other states [β = -0.836, t_2_ = -0.773, p = 0.44]. When compared to an annual income < $15k, those with an annual income >$150k [β = 4.602, t_6_ = 2.716, p = 0.007] scored higher. This effect was not seen in the other income comparisons. Higher scores were seen with those who did not list radio [β = 2.885, t_1_ = 2.668, *p* = 0.008] or social media [β = 2.948, t_1_ = 2.455, *p* = 0.014] as one of their top three sources of information than those who did. This effect was not seen in the other sources of information. The adjusted R^2^ for this model was 5.9% [p < 0.001].

### Practices

There were no differences based on state. Compared to females, males had worse practices [β = -3.54, t_2_ = -4.316, *p* < 0.001] and non-binary individuals did not differ in practices [β = -1.572, t_2_ = -0.991, *p* = 0.322]. Essential compared to non-essential workers [β = -3.152, t_1_ = -3.447. *p* = 0.001] and non-healthcare workers compared to healthcare workers [β = -2.509, t_1_ = -.205, *p* = 0.04] scored lower. People who did not utilize newspapers [β = -3.135, t_1_ = -3.674, *p* < 0.001], scholarly articles [β = -2.879, t_1_ = -3.136, *p* = 0.002], or television [β = -2.34, t_1_ = -2.78, *p* = 0.006] as a top three primary information source scored lower compared to those who did. No differences were seen in other sources of information for users who endorsed utilizing that medium versus those who did not. Those who lived in an apartment [β = -2.261, t_2_ = -2.69, *p* = 0.007] or those who indicated an alternative living arrangement [β = -5.717, t_2_ = -2.124, *p* = 0.034] scored lower compared to those in a house. The adjusted R^2^ for this model was 19.9% [p < 0.001].

## Discussion

To the best of our knowledge, this is the first paper comparing U.S. COVID-19 KAP focused on two of the most affected states at the onset of the pandemic, California and New York. Specifically, our diverse, cross-sectional sample adequately represents the population of San Francisco Bay Area and New York Metropolitan Area social media users. Most had good knowledge and practices, defined as > 80%, but had a poor attitude, defined at < 80%. Demographic differences were predictive of higher or lower scores in each KAP section. We identified specific weaknesses in public health information dissemination and use of social media to obtain information emerged as a salient theme. In accordance with a preliminary systematic review of COVID-19 KAP across the United States conducted by Sarria-Guzmán et al., our study supports that improved educational resources should target disadvantaged populations and communities, as vulnerable socioeconomic groups demonstrate less knowledge about COVID-19 [26]. There was no association between attitude and practices but there was a positive association between attitude and knowledge as well as knowledge and practices.

### Knowledge

The answers to knowledge questions were based on contemporary CDC findings [4/20/20–5/22/20], suggesting that dissemination of public health information was successful. Statistically significant predictors of greater knowledge about COVID-19 include Caucasian race, non-essential worker status, and California residence. Our data supports previous literature that suggests a failure of public health initiatives to accommodate people with lower health literacy [27]. Those with higher education backgrounds had higher knowledge scores than those who finished high school or less, suggesting that health guidelines and information during a pandemic should be simplified to accommodate lower health care literacy levels [15, 16, 26]. Furthermore, our study found that ethnic minorities had lower knowledge scores. Compared to previous surveys on COVID-19 knowledge, which only listed African American and Hispanic individuals as having lower scores, we found that those who identified as Asian or as mixed race also had lower average knowledge scores when controlling for income and education [20].

Even when substratifying for similar levels of income and education, implicit and structural racism impact the quality of healthcare and public health education for non-white minorities [21]. This detriment in quality may contribute to understanding of public health knowledge [21]. Since disparities in COVID-19 knowledge reception have been postulated as a mechanism by which COVID-19 disproportionately affects minority populations, it is necessary for public health campaigns to address the needs of these populations through translated materials, collaboration with trusted community leaders, and increased efforts to understand cultural barriers in knowledge reception. Furthermore, if research demonstrates knowledge distribution of equitable quality, other considerations of knowledge reception must be examined. For instance, non-white communities have a long history of mistrust towards medical and government institutions [28, 29]. A causal analysis of mistrust towards these institutions would better identify factors associated with increased trust and acceptance of public health knowledge. This analysis could better elucidate if the solution arises from addressing previously postulated mechanisms such as increased non-white representation in those distributing public health information or if there are other mechanisms at play that have not yet been considered by the scientific community.

Participants in New York, which had more cases and a higher rate of infection than California at the time the survey was conducted, had worse knowledge about the virus [24]. Regional disparities in knowledge may suggest varying effectiveness of local public health campaigns. A thorough analysis of New York and California public health information distribution may highlight discrepancies that contributed to differences in COVID-19 cases between the two states. This finding would be useful in assessing the efficacy of public health communication strategies regarding the COVID-19 pandemic and for other public health crises. Alternatively, the regional distribution of misinformation campaigns may correlate with low knowledge scores. In a Canadian study by Parsons et al., regions with higher caseloads, hospitalizations, and fatalities were associated with susceptibility to misinformation campaigns [30]. Misinformation campaigns are often circulated on the internet and social media [30]. Our study had a similar proportion of internet and social media users from both California and New York; therefore, we cannot confirm that misinformation is associated with our regional knowledge differences. An analysis of misinformation spread via Twitter during May and June 2020 demonstrated that New York and California had comparable numbers of misinformation tweets per 1000 residents, despite New York having a higher rate of cases and hospitalizations during the same period [31]. However, many misinformation studies are limited by the complexity of analyzing social media posts by region and the ease of interregional spread of misinformation, suggesting that the proportion of misinformation campaigns spread throughout a particular region may be underestimated. A systematic review of KAP studies during COVID-19 corroborates the importance of community level educational initiatives in improving knowledge [32].

Knowledge scores were lowest in categories such as virus etiology, risk factors, and symptoms. Prior studies suggest that the spread of misinformation contributed to deficits in accurate knowledge of COVID-19 [31, 33]. Our paper investigated differences in knowledge based on primary sources of information and found that those who utilized newspapers had higher knowledge scores while those utilizing sources identified as “other” had lower knowledge scores. We found no significant difference between source of information and knowledge score for those who used the internet and social media versus other mediums, likely due to our method of sampling. Generally, scholarly journals and newspaper articles are filtered and vetted; therefore, they are less likely to be associated with misinformation. In our study, similar to other KAP studies, more than half of the participants depended on the internet and social media as a primary source of information [8–14]. Hassan et al. [2016] found that during the MERs-CoV outbreak, nearly half of the participants who utilized the internet believed that their information was accurate [12]. Our participants were internet and social media users, and therefore were likely to come by information about the virus via the internet. It is possible that survey respondents would have higher knowledge about public health based on their willingness to respond; however, given the cross-sectional approach and breadth of advertising on diverse Facebook groups, the authors expect that a representative sample of social media users in the San Francisco Bay Area and New York Metropolitan Area was obtained.

Media trust and selective exposure theory postulates that people with underlying government skepticism may seek out misinformation to confirm their beliefs [33, 34]. In addition to efforts that aim to combat misinformation [e.g. consistent public health updates, algorithms to identify and flag misinformation] pertinent to preventing future pandemic fatalities, it is also important to address skepticism and distrust [35]. Furthermore, our study identifies viral etiology, COVID-19 risk factors, and COVID-19 symptoms as categories of knowledge that should be targeted in efforts to control misinformation in public health education campaigns.

### Attitudes

Despite high knowledge scores, low attitude scores reflect pessimistic beliefs surrounding the pandemic and the future. Statistically significant predictors of attitude included gender, income, state of residence, and information source. Although strong emotions such as fear can imbue memories with heightened salience and improve recall up to a certain threshold, overwhelming emotion can prevent deep encoding of memories and application of knowledge to practices [36]. Almutairi et al. [2012] found that the emotional impact of MERS-CoV improved precautionary measures taken by participants [11]. In contrast, this study did not find a strong correlation between attitudes and practices or knowledge.

Low attitude scores could be a result of many factors, including the novelty and global nature of the virus as well as inconsistent messaging from political leadership [37]. A study amongst Chinese social media users found that clear information in the news, education regarding the necessity of social distancing and travel restriction, and availability of convenient and accessible medical care was associated with improved attitude [38]. To improve public attitude, healthcare systems should ensure that the public can easily access clear, consistent information. Additionally, improved transparency regarding accessibility to medical care during the pandemic may lessen the anxiety around needing medical attention. This may be achieved by frequently updating emergency room or clinic wait times and appointment availability for hospital systems online.

Our study supports findings in recent literature regarding pandemics that women had more negative attitudes regarding COVID-19 than men [39]. Though several systematic reviews regarding COVID-19 KAP studies demonstrate KAP differences based on gender, the mechanism behind this association is not commonly discussed [32, 40]. The authors of this study postulate that COVID-19 has had a significant impact on gender equality. In response to daycare and school closures, working mothers decreased their work hours four to five times more than fathers. This phenomenon has increased the gender gap in work hours by 20–50%, leaving female employment vulnerable to a loss of health insurance or job insecurity [41]. Additionally, as women more frequently are tasked with the role of primary care provider within a household, they are more likely to have higher risks of exposure and a greater burden of caring for people in the midst of quarantine restrictions [39]. Women also tend to believe that COVID-19 poses a serious health risk more than men, suggesting that worse attitudes may in part be related to concern about virus related hospitalization and death [42, 43]. This difference has been seen on a larger scale, as multiple countries whose elected leaders are women have been praised for their caution and effective response to the pandemic. On the contrary, multiple countries whose elected leaders are men, such as the United States and Brazil, were slower to recognize the severity of the virus and adopt national preventative measures [42, 44]. Despite this difference, fatality rates by country have been similar, regardless of the gender of elected leaders. However, studies have been limited by low numbers of women in leadership positions and the tendency of women and men in leadership roles to over and underestimate deaths, respectively [44]. Further analysis regarding the role of gender differences in relation to mitigating COVID-19 adverse effects should consider increased recognition of virus-related health risks and adopt cautionary measures as potential mediators.

Participants from New York had lower attitude scores in comparison to those in California, suggesting that at the time of data collection both lower knowledge and worse attitudes were found in the region with the higher case load. Whereas optimism has been associated with proactive behavior, worse attitudes predict less engagement with health-protective behaviors [45]. Regional geographic differences such as city planning and the proportion of urban to suburban areas have been shown to be significant predictors of KAP in systematic reviews of COVID-19 [26]. Further research is needed to determine if the worse attitudes were a result of fear and anxiety associated with living in an area with a higher case load, or if having increased negativity and anxiety resulted in decreased adherence to public health guidelines. Perhaps adherence to preventative public health strategies would improve if physicians and community leaders focused on encouraging optimism, and if policymakers funded programs that provide free mental health services.

Those who received information primarily from social media had lower attitude scores, consistent with known associations between social media and anxiety [46]. The internet and social media provide many individuals with accessible information about public health guidelines and regulations. Yet, even minimal exposure to pandemic news on social media has been associated with negative emotional consequences [47]. To maintain the benefits of internet and social media exposure while limiting anxiety, experts have called for positive information and easily digestible guidelines to be integrated into pandemic news [47]. Until then, prescriptions for mental health care may include becoming unplugged.

### Practices

The average respondent had positive health-protective practices. Lunn et al. [2020] reported that people are more likely to engage in altruistic behavior if the actions of their community align with the behavior [5]. Our study investigated the association between demographic variables and practices, but not if behavior was directly impacted by the opinions and actions of those in their community. It is, however, important to recognize that social networks and communities often share characteristics that can be identified by the demographic variables we studied. Therefore, these variables may have potential interrelation with the influence of one’s community on behavior. Significant predictors of practice scores included gender, status as an essential or healthcare worker, and information source.

Interestingly, while a majority of participants endorsed social distancing, mask wearing, and handwashing, only slightly above 60% indicated that they eat a healthy diet or live a healthy lifestyle. The role of nutrition, management of chronic disease, and inflammation has significantly influenced COVID-19 disease progression [48]. Despite these benefits, dietary and lifestyle change is difficult and expensive. Behavioral change is more likely when the modification is easy [5]. While hand washing is easier than healthy eating, physicians should emphasize modification of lifestyle factors and preventative health efforts such as quitting smoking, drinking less alcohol, and exercising daily, as these factors play a role in general health as well as COVID-19 disease progression.

Women had higher practice scores compared to men. This finding has been reported in relation to greater health-protective behaviors during the COVID-19 pandemic such as mask usage [43]. The discrepancy in practice scores could be because women, despite political affiliation, are less likely to subscribe to COVID-19 misinformation campaigns than men [49]. Pandemic behavioral analysis corroborates the finding that women are 50% more likely than men to subscribe to non-pharmaceutical behaviors and have more characteristics, such as agreeableness and conscientiousness, that are associated with compliance with public health behaviors [50, 51]. It has also been postulated that women, who traditionally assume caregiving roles, are more likely to view protective behaviors such as mask wearing as a necessary precaution to keep their family safe [43, 50]. Practice differences are clinically significant as men have been more likely to be infected with and experience complications from COVID-19 [43, 50, 51]. Prior studies in Saudi Arabia suggest that providing information regarding respiratory viruses to married women enables the women to subsequently distribute this information to their husbands [10, 11]. In our sample population, the majority of participants [62.07%] were not married and likely abided by different cultural customs regarding gender normative behavior. Thus, it may be beneficial for public health campaigns to target men, encourage self-protective behaviors, and unravel misconceptions surrounding masculinity and mask wearing [43].

Furthermore, while we predicted essential workers would be more adherent to public health guidelines, essential workers had worse practices than non-essential workers. This finding could be explained by factors such as inadequate PPE provision, an inability to social distance at work, inadequate training on PPE use, inconsistent requirements on PPE donning, inconsistencies between national and local guidelines, and lack of mandatory training specific to infectious disease prevention [52, 53]. Understanding that essential workers had worse practices and a higher likelihood of engaging in behaviors that allow them to spread and contract COVID-19, federal and state policymakers should provide employers with incentives for implementing social distancing policies, providing adequate PPE, and allowing for paid sick leave for COVID-19 positive patients. In contrast, healthcare workers, although essential, had better practices than non-healthcare workers. While this may in part explain their increased occupational risk of contracting the virus, improved knowledge during infectious outbreaks has been correlated with improved practices [4, 8, 54, 55]. Unsurprisingly, healthcare workers had higher knowledge scores compared to those who did not work in the healthcare field.

There were also differences in practices based on participants’ utilized source of information. Recognizing that education influences prevention, it is crucial that public health campaigns distribute information across a range of mediums for improved practices. Additionally, an emphasis on concise language and reputable facts that refute the spread of misinformation should be implemented, as recent studies demonstrate that the method of information dissemination is critical to how it is applied [55]. Although our study did not discriminate between sources that were locally or federally based, inconsistencies and ambiguities between local and federal public health guidelines were cited as a reason for poor adherence to public health guidelines in prior studies [53]. In that vein, education about COVID-19 should not be purely epidemiological but also should include social learning with extreme efforts made to minimize misinformation.

### Limitations

The survey was distributed online to limit in-person contact during the pandemic. As participants from California and New York were recruited via social media, it is possible that KAP may be influenced by the social, political, and ideological leanings of these geographic locations, and thus may not be generalizable to the United States at large.

Furthermore, data collection spanned several weeks, occurring between 4/20/20 and 5/22/20. Since the time of data collection, information about the virus became more readily available, increasing access to knowledge about the pandemic and public health behaviors. Additionally, the ongoing and shifting nature of the pandemic has likely influenced the KAP of individuals across the United States and across the world. There was no availability of COVID-19 vaccines nor had any SAR-CoV-2 variants been discovered or brought to public attention at the time of data collection [25]. In addition, the lasting and erratic nature of COVID-19 surges since the onset of the pandemic has had negative psychological consequences and has contributed to pandemic fatigue, defined by the World Health Organization as “demotivation to follow recommended protective behaviors,” which may be variably impacting different demographic groups [56, 57]. Additional KAP assessments specific to COVID-19 vaccines, variants, and non-quarantine lifestyles would be extremely beneficial to current public health campaigns.

## Supporting information

S1 Appendix Questionnaire breakdown and response percentages. (PDF)

S2 Appendix Questionnaire scoring guidelines and exemptions. (DOCX)

S3 Appendix KAP univariate analysis. (XLSX)
