# Supplementary material for: Knowledge, attitudes, and practices [KAP] toward COVID-19: A cross-sectional study in the New York Metropolitan Area and California Bay Area
Source: PLoS One. 2022 Aug 10;17(8):e0271212. doi: 10.1371/journal.pone.0271212 (PMC9365154; doi:10.1371/journal.pone.0271212)
Supplement: S1 Appendix — (PDF) [file pone.0271212.s001.pdf]

Question #	Knowledge	No No. (%)	Not Sure No. (%)	Yes No. (%)
1	Is COVID-19 caused by a virus?	3 (0.40)	19 (2.80)	653 (96.70)
2	Is COVID-19 caused by bacteria?	596 (88.30)	41 (6.10)	38 (5.60)
3	Is COVID-19 an inherited disease?	653 (96.70)	17 (2.50)	5 (0.70)
4	Can COVID-19 spread from person to person?	2 (0.30)	2 (0.30)	671 (99.40)
5	Can COVID-19 be spread by contaminated surfaces and objects?	4 (0.60)	27 (4.00)	644 (95.40)
6	Can COVID-19 be spread by droplets after coughing or sneezing?	1 (0.10)	2 (0.30)	672 (99.60)
7	Can COVID-19 be spread by talking to an infected person from less than 6 feet away?	15 (2.20)	47 (7.00)	613 (90.80)
8	Can COVID-19 take up to 14 days after an infection to cause symptoms?	4 (0.60)	21 (3.10)	650 (96.30)
9	Can COVID-19 cause the same symptoms as the seasonal flu (fever, cough, sore throat, muscle aches)?	11 (1.60)	9 (1.30)	655 (97.00)
10	Can COVID-19 cause pneumonia?	23 (3.40)	102 (15.10)	550 (81.50)
11	Can COVID-19 cause Severe Acute Respiratory Distress Syndrome?	22 (3.30)	87 (12.90)	566 (83.90)
12	Can some people infected with COVID-19 have no symptoms?	2 (0.30)	9 (1.30)	664 (98.40)
13	Are elderly at a higher risk of serious disease from COVID-19?	3 (0.40)	3 (0.40)	669 (99.10)
14	Are young children at a higher risk of serious disease from COVID-19?	420 (62.20)	156 (23.10)	99 (14.70)
15	Does having a pre-existing condition (such as diabetes or hypertension) put someone at higher risk of serious illness from COVID-19?	6 (0.90)	13 (1.90)	656 (97.20)
16	Are cancer patients at a higher risk of serious disease from COVID-19?	7 (1.00)	101 (15.00)	567 (84.00)
17	Are teenagers at a higher risk of serious disease from COVID-19?	511 (75.70)	142 (21.00)	22 (3.30)
18	Are people on medication that suppresses their immune system at a higher risk of serious disease from COVID-19?	2 (0.30)	42 (6.20)	631 (93.50)
19	Is a vaccine available for COVID-19?	656 (97.20)	15 (2.20)	4 (0.60)
20	Are you protected from COVID-19 by your annual influenza vaccine?	651 (96.40)	18 (2.70)	6 (0.90)
21	Is there a cure for COVID-19?	640 (94.80)	28 (4.10)	7 (1.00)
22	Can people die from COVID-19?	2 (0.30)	2 (0.30)	671 (99.40)
23	Can young people die from COVID-19?	5 (0.70)	9 (1.30)	661 (97.90)
24	Did COVID-19 first come from humans?	517 (76.60)	142 (21.00)	16 (2.40)
25	Did COVID-19 first come from bats?	48 (7.10)	297 (44.00)	330 (48.90)
26	Was the agent that causes COVID-19 made by humans in a scientific laboratory?	412 (61.00)	225 (33.30)	38 (5.60)

Question #	Attitude	Strongly disagree No. (%)	Somewhat disagree No. (%)	Neither agree nor disagree No. (%)	Somewhat agree No. (%)	Strongly agree No. (%)
1	I think social distancing is important to keep myself and others disease-free.	3 (0.40)	10 (1.50)	5 (0.70)	47 (7.00)	610 (90.40)
2	I think social distancing is necessary to keep myself and others disease-free.	5 (0.70)	9 (1.30)	8 (1.20)	66 (9.80)	587 (87.00)
3	I know how to protect myself from COVID-19.	0 (0.00)	7 (1.00)	16 (2.40)	281 (41.60)	371 (55.00)
4	I can trust federal government guidelines to tell me how to protect myself from COVID-19.	114 (16.90)	139 (20.60)	102 (15.10)	264 (39.10)	56 (8.30)
5	I can trust state government guidelines to tell me how to protect myself from COVID-19.	33 (4.90)	49 (7.30)	57 (8.40)	291 (43.10)	245 (36.30)
6	I am afraid of getting COVID-19 from a family member.	145 (21.50)	142 (21.00)	173 (25.60)	157 (23.30)	58 (8.60)
7	I am afraid of getting COVID-19 from a friend.	79 (11.70)	108 (16.00)	150 (22.20)	233 (34.50)	105 (15.60)
8	I am afraid of getting COVID-19 at work.	136 (20.10)	54 (8.00)	168 (24.90)	137 (20.30)	180 (26.70)
9	I am afraid of going to common places (grocery store, school, work, etc.).	48 (7.10)	71 (10.50)	91 (13.50)	271 (40.10)	194 (28.70)
10	I worry about the economy.	11 (1.60)	19 (2.80)	45 (6.70)	206 (30.50)	394 (58.40)
11	I worry about getting or keeping a job.	98 (14.50)	93 (13.80)	118 (17.50)	177 (26.20)	189 (28.00)
12	It is important to wear a face mask while in common spaces.	7 (1.00)	15 (2.20)	18 (2.70)	91 (13.50)	544 (80.60)
13	COVID-19 is preventable.	24 (3.60)	76 (11.30)	146 (21.60)	307 (45.50)	122 (18.10)
14	I worry that a family member or friend will get sick.	5 (0.70)	29 (4.30)	38 (5.60)	177 (26.20)	426 (63.10)
15	I have not learned about disease prevention practices such as social distancing before.	237 (35.10)	139 (20.60)	62 (9.20)	133 (19.70)	104 (15.40)
16	I worry about getting enough food.	264 (39.10)	188 (27.90)	83 (12.30)	111 (16.40)	29 (4.30)
17	I worry about the shortages of needed supplies.	58 (8.60)	113 (16.70)	52 (7.70)	279 (41.30)	173 (25.60)
18	If I got sick, I think the hospital could take care of me.	33 (4.90)	112 (16.60)	103 (15.30)	316 (46.80)	111 (16.40)
19	I am able to work from home.	95 (14.10)	32 (4.70)	83 (12.30)	121 (17.90)	344 (51.00)
20	If I got COVID-19, I think I would be okay.	38 (5.60)	108 (16.00)	182 (27.00)	246 (36.40)	101 (15.00)
21	Online or phone contact is just as good as in-person contact.	242 (35.90)	235 (34.80)	64 (9.50)	100 (14.80)	34 (5.00)
22	I have had trouble sleeping in the past week.	142 (21.00)	84 (12.40)	72 (10.70)	207 (30.70)	170 (25.20)
23	I feel hopeful about the future.	61 (9.00)	152 (22.50)	113 (16.70)	248 (36.70)	101 (15.00)
24	I feel lonely.	113 (16.70)	129 (19.10)	95 (14.10)	221 (32.70)	117 (17.30)
25	I feel nervous.	77 (11.40)	88 (13.00)	73 (10.80)	265 (39.30)	172 (25.50)
26	If I could, I would get vaccinated against COVID-19.	28 (4.10)	21 (3.10)	35 (5.20)	80 (11.90)	511 (75.70)
27	I have a clear understanding of what "social distancing" means.	4 (0.60)	2 (0.30)	6 (0.90)	89 (13.20)	574 (85.00)
28	If there is no vaccine and we stop social distancing, most people will become infected with COVID-19.	12 (1.80)	17 (2.50)	82 (12.10)	237 (35.10)	326 (48.30)
29	The economy will open up within the next three months.	71 (10.50)	134 (19.90)	188 (27.90)	205 (30.40)	77 (11.40)

Question #	Practices	No No. (%)	Sometimes No. (%)	Yes No. (%)
1	I wash my hands throughout the day	14 (2.10)	53 (7.90)	608 (90.10)
2	I use soap and water to wash my hands continuously.	30 (4.40)	51 (7.60)	594 (88.00)
3	I cover my nose and mouth with a tissue during sneezing or coughing.	50 (7.40)	130 (19.30)	495 (73.30)
4	I throw used tissue in the trash.	9 (1.30)	34 (5.00)	632 (93.60)
5	I avoid touching my eyes, nose, or mouth as much as I can.	37 (5.50)	189 (28.00)	449 (66.50)
6	I use face masks in crowds.	14 (2.10)	36 (5.30)	625 (92.60)
7	I eat healthy.	23 (3.40)	197 (29.20)	455 (67.40)
8	I live a healthy lifestyle.	32 (4.70)	226 (33.50)	417 (61.80)
9	I sanitize surfaces in my home weekly.	132 (19.60)	159 (23.60)	384 (56.90)
10	I avoid leaving my home when possible.	27 (4.00)	73 (10.80)	575 (85.20)
11	I avoid travel for leisure right now.	17 (2.50)	28 (4.10)	630 (93.30)
12	If I felt flu-like symptoms, I would self-isolate.	4 (0.60)	16 (2.40)	655 (97.00)
13	I limit my in-person interactions with others.	10 (1.50)	35 (5.20)	630 (93.30)
14	I interact with someone who has COVID-19 in-person.	636 (94.20)	17 (2.50)	22 (3.30)
15	I work from home.	178 (26.40)	45 (6.70)	452 (67.00)
16	I watch or read the news a lot.	96 (14.20)	161 (23.90)	418 (61.90)
17	I have been social distancing since March 20th.	20 (3.00)	30 (4.40)	625 (92.60)
18	I have been social distancing since April 3rd.	51 (7.60)	21 (3.10)	603 (89.30)
19	I have been social distancing since April 17th.	51 (7.60)	21 (3.10)	603 (89.30)
20	I have been social distancing since May 1st.	358 (53.00)	19 (2.80)	9 (1.30)
