# Supplementary material for: Knowledge, attitudes, and practices [KAP] toward COVID-19: A cross-sectional study in the New York Metropolitan Area and California Bay Area
Source: PLoS One. 2022 Aug 10;17(8):e0271212. doi: 10.1371/journal.pone.0271212 (PMC9365154; doi:10.1371/journal.pone.0271212)
Supplement: S2 Appendix — (DOCX) [file pone.0271212.s002.docx]

**Appendix B. Questionnaire Scoring Guidelines and Exemptions**

The survey was available in both English and Spanish. It began with 17 demographic questions. Answers to the questions in each KAP section were scaled in order to create a standardized range from 0-100 for knowledge, attitudes, and practices sections separately.

**Demographic Questions**

Participants were limited to one response for the majority of the questions, with the exception of Questions 6, 9, and 15. Question 9 was answered by selecting a state of residence from a drop-down menu, which included all 50 states, the District of Columbia, and “International” as answer choices. Multiple responses could be selected for question 6 and question 15, although both questions instructed participants to select up to three responses.

**Knowledge Questions**

Knowledge questions were scored so that 3 points were assigned for the correct answer, 1 point for the incorrect answer, and 2 points if the participant indicated “not sure.” Correct answers were based on CDC guidelines published as of 4/15/20. Question #5 was excluded from contributing to the total knowledge score because of changing guidelines recommended by the CDC. The scaled score was calculated using the following equation: (-2425 + 99 x Survey Score)/50. A minimum survey score of 20 resulted in a scaled score of 1 and a maximum survey score of 60 resulted in a scaled score of 100.

**Attitude Questions**

Attitude was measured on a 5-point Likert scale of agreement. A positive attitude, indicating a low level of anxiety or worry, was given a 5, while a negative attitude was given a 1. Questions 26, 28, and 29 were excluded from the aggregate attitude score because they were meant to survey beliefs rather than attitudes. The scaled score was calculated using the following equation: (-2470 + 99 x Survey Score)/104. A minimum survey score of 26 resulted in a scaled score of 1 and a maximum survey score of 130 resulted in a scaled score of 100.

**Practice Questions**

Participants answered questions of practice by selecting “yes,” “no,” or “sometimes” as a response. Practices were scored such that 3 points were given for proper practices and 1 point for improper practices. An answer of “sometimes” was assigned 2 points. Questions about recommended practices were taken from CDC recommendations. Responses to the practices section were scaled using two separate equations so that all participants had a practice score ranging from 0-100. Question number 20 of the practices section was made active after May 1st, 2020. Responses to questions 15 and 16 were frequently incomplete or ambiguous in whether they were considered “good,” so both questions were excluded from the calculated practice scores. Although it is beneficial to remain informed about updated guidelines, social media use has been shown to increase negative thoughts and rumination, and therefore use of social media may be interpreted as an ambiguous or negative practice.[^24^](https://www.zotero.org/google-docs/?61vK18) Prior to 5/1/2020, the scaled 17 question score was calculated using the following equation: (-1649 + 99 x Survey Score)/34. A minimum survey score of 17 resulted in a scaled score of 1 and a maximum survey score of 51 resulted in a scaled score of 100. After 5/1/2020, the scaled 18 question score was calculated using the following equation: (-1746 + 99 x Survey Score)/36. A minimum survey score of 18 resulted in a scaled score of 1 and a maximum survey score of 54 resulted in a scaled score of 100.
